# Lattice Chloride Shielding and Reconstructed Phosphate Armor for Stable Seawater Electrolysis

**DOI:** 10.1002/advs.77420

**Published:** 2026-08-25

**Authors:** Yatao Yan, Yuxing Lin, Xing Chen, Shuo Yang, Ke Zhou, Jifang Chen, Lubin Ni, Ming Chen

**Affiliations:** ^1^ College of Chemistry and Materials Yangzhou University Yangzhou People's Republic of China; ^2^ Department of Physics Xiamen University Xiamen People's Republic of China; ^3^ Institute of Technology for Carbon Neutrality Shenzhen Institutes of Advanced Technology Chinese Academy of Sciences Shenzhen People's Republic of China; ^4^ Anhui Provincial Key Laboratory of Green Carbon Chemistry School of Chemistry and Materials Engineering Fuyang Normal University Fuyang People's Republic of China; ^5^ Jiangsu Provincial Key Laboratory of Green & Functional Materials and Environmental Chemistry Yangzhou University Yangzhou People's Republic of China

**Keywords:** chloride corrosion resistance, dual‐protection mechanism, halogen regulation, seawater electrolysis, surface reconstruction

## Abstract

Achieving selective oxygen evolution while suppressing competing chlorine evolution and chloride‐induced corrosion remains a key challenge for practical seawater electrolysis. Herein, a series of halogen‐modified heterostructures, X‐Co(OH)_2_@CoP_4_ (X = F, Cl, Br, I), are constructed via an electrochemical synthesis strategy, in which Cl‐Co(OH)_2_@CoP_4_ exhibits the optimal catalytic performance following a volcano‐type activity trend. In situ and ex situ analyses reveal that lattice Cl incorporation coupled with reconstructed phosphate species establishes a dual‐protection mechanism that promotes selective OH^−^ adsorption while suppressing competing chlorine evolution reactions during seawater electrolysis. Specifically, lattice Cl regulates the electronic structure of Co centers and suppresses chloride‐induced corrosion, while the surface CoP_4_ layer undergoes in situ transformation into phosphate species under anodic polarization, generating a protective outer layer. Density functional theory calculations further confirm that halogen incorporation enhances OH^−^ selectivity and lowers the reaction free‐energy change. Benefiting from this cooperative regulation strategy, a symmetric Cl‐Co(OH)_2_@CoP_4_ electrolyzer delivers a current density of 500 mA cm^−2^ at only 1.67 V in alkaline seawater and operates stably for over 2700 h. This work provides an effective strategy for constructing corrosion‐resistant electrocatalysts that integrate intrinsic electronic regulation with dynamic surface protection for practical seawater electrolysis.

## Graphical abstract

A dual‐protection strategy integrating lattice Cl shielding with dynamically reconstructed phosphate armor enables selective OH adsorption and suppresses chloride‐induced corrosion during alkaline seawater electrolysis, delivering durable operation at industrial‐level current densities.

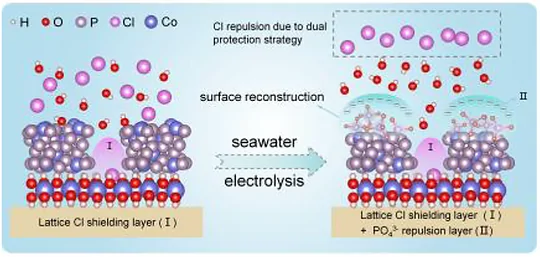

## 1 Introduction

Electrochemical water splitting is widely regarded as one of the most promising technologies for producing green hydrogen and is expected to play a central role in the future hydrogen economy. However, conventional water electrolysis systems rely heavily on high‐purity freshwater resources, which limits their large‐scale deployment in regions facing freshwater scarcity [1, 2]. In contrast, direct seawater electrolysis provides an attractive alternative owing to the abundance and accessibility of seawater resources, particularly in coastal areas, offering a sustainable pathway for large‐scale hydrogen production while alleviating freshwater consumption [3, 4, 5]. Nevertheless, the complex composition of seawater, especially the high concentration of chloride ions, introduces significant challenges for practical electrolysis applications. During anodic oxidation, chloride ions can participate in competing chlorine evolution reactions (ClER) [6, 7], which not only reduce the selectivity toward the oxygen evolution reaction (OER) but also generate corrosive chlorine‐containing species that accelerate catalyst degradation and electrode failure [8, 9]. Moreover, chlorine species produced at high anodic potentials can dissolve and hydrolyze in the electrolyte, further increasing local acidity and aggravating structural instability of catalytic materials [10, 11, 12]. Therefore, developing highly active and corrosion‐resistant OER electrocatalysts remains essential for achieving efficient and durable seawater electrolysis.

Notably, previous studies have demonstrated that the thermodynamic potential difference between OER and ClER can reach approximately 480 mV in alkaline electrolytes (pH > 7.5), suggesting that selectively promoting OER kinetics while suppressing chloride oxidation is feasible through rational catalyst design [13, 14]. Layered double hydroxides (LDHs) have attracted extensive attention as promising OER electrocatalysts due to their tunable metal composition and flexible interlayer chemistry [12, 15]. For example, constructing atomic Ir sites on CoFe‐LDH can regulate Cl^−^ adsorption via an Ir–OH/Cl coordination environment, thereby lowering OER activation energy and mitigating chloride‐induced corrosion during alkaline seawater electrolysis [16]. Similarly, surface modification of layered double hydroxides (LDHs) with oxyanions (e.g., SO_4_^2−^, CO_3_^2−^, and PO_4_^3−^) can generate chloride‐repelling environments that enhance catalytic stability during seawater electrolysis [17, 18, 19]. However, the intrinsic lamellar structure of LDHs remains vulnerable to chloride corrosion and metal ion dissolution during long‐term operation, leading to gradual degradation of catalytic performance [20, 21, 22]. Constructing protective surface layers has therefore emerged as an effective strategy to improve the structural stability of LDH‐based catalysts [23]. For instance, anchoring PW_12_ polyoxometalates on CoFe hydroxide anodes can regulate Cl^−^/OH^−^ adsorption and stabilize metal active sites, thereby suppressing chlorine reactions and improving corrosion resistance [24]. Nevertheless, single modification strategies such as elemental doping, interlayer anion regulation, or surface coating alone are often insufficient to simultaneously optimize catalytic activity and corrosion resistance under high‐current‐density seawater electrolysis conditions [25]. Recent studies further indicate that halogen doping can effectively regulate the electronic distribution of transition‐metal centers [26], optimize adsorption energies of oxygen‐containing intermediates [21, 27], and suppress competitive chloride adsorption on catalytic surfaces, providing a promising pathway for improving both OER activity and corrosion resistance in seawater electrolysis systems. However, the synergistic integration of internal halogen regulation and dynamically reconstructed surface protection layers for simultaneously enhancing catalytic selectivity and structural durability remains insufficiently explored.

In this work, we report a Cl‐Co(OH)_2_@CoP_4_ heterostructured electrocatalyst featuring lattice chloride regulation coupled with dynamically reconstructed phosphate protection for efficient alkaline seawater electrolysis. Lattice Cl incorporation modulates the electronic structure of catalytic Co centers and suppresses chloride‐induced corrosion, while the surface CoP_4_ layer undergoes in situ transformation into phosphate species under anodic polarization, forming a protective outer layer that stabilizes catalytic active sites during operation. Notably, a systematic comparison of halogen‐modified catalysts (X = F, Cl, Br, I) reveals a volcano‐type activity relationship, indicating that lattice halogen incorporation plays a decisive role in regulating catalytic selectivity and reaction kinetics under seawater electrolysis conditions. Density functional theory calculations further demonstrate that halogen incorporation weakens chloride adsorption while lowering the reaction free‐energy changes for both hydrogen and oxygen evolution reactions, thereby confirming the critical role of lattice halogen regulation in promoting selective OH^−^ adsorption and suppressing competing chlorine evolution reactions. Benefiting from this cooperative regulation strategy, the symmetric Cl‐Co(OH)_2_@CoP_4_ electrolyzer delivers a low cell voltage of 1.67 V at 500 mA cm^−2^ with stable operation over 2700 h in alkaline seawater, highlighting its strong potential for practical seawater electrolysis applications.

## 2 Results and Discussion

### 2.1 Catalyst Synthesis and Physical Characterizations

The synthesis procedure of Cl‐Co(OH)_2_@CoP_4_ is schematically illustrated in Figure 1a. First, a dense array of Cl‐Co(OH)_2_ nanosheets was electrodeposited on a titanium mesh substrate at −0.7 V for 90 min (Figure S1). In contrast, the non‐doped Co(OH)_2_ nanosheets exhibit a noticeably thinner and curved morphology (Figure S2), indicating that chloride ions in the electrolyte play an important role in regulating nanosheet growth during electrodeposition. This structural difference is further confirmed by transmission electron microscopy (TEM) observations, which clearly reveal the distorted morphology of pristine Co(OH)_2_ nanosheets and the relatively flat and well‐defined nanosheet structure of Cl‐Co(OH)_2_ (Figure S3). Further investigation shows that increasing the chloride concentration within the studied range (KCl: 0.05 m∼0.2 m) does not significantly alter the nanosheet morphology (Figure S4), suggesting that the growth‐regulating effect of chloride ions is established at an early stage of nucleation and maintained during subsequent nanosheet evolution. Subsequently, CoP_4_ nanoparticles were electrodeposited on the surface of the Cl‐Co(OH)_2_ nanosheets from an electrolyte containing Co^2+^ and NaH_2_PO_2_, forming a hierarchical heterostructure. The deposition time plays a critical role in regulating the surface coverage and structural integration of the CoP_4_ layer. As shown in Figure 1b, short deposition times lead to sparse CoP_4_ nanoparticles on the nanosheet surface, which are insufficient to provide effective protection for the underlying active layer. When the deposition time is increased to 60 min, uniformly distributed CoP_4_ nanoparticles fully cover the Cl‐Co(OH)_2_ nanosheets, forming a porous surface architecture that facilitates electrolyte penetration and gas release during electrochemical reactions (Figure 1c). However, excessive deposition results in the formation of large CoP_4_ particles, which reduces the exposed active surface area and increases the electron transport distance between CoP_4_ and Cl‐Co(OH)_2_, thereby hindering interfacial charge transfer (Figure 1d).

**FIGURE 1:**
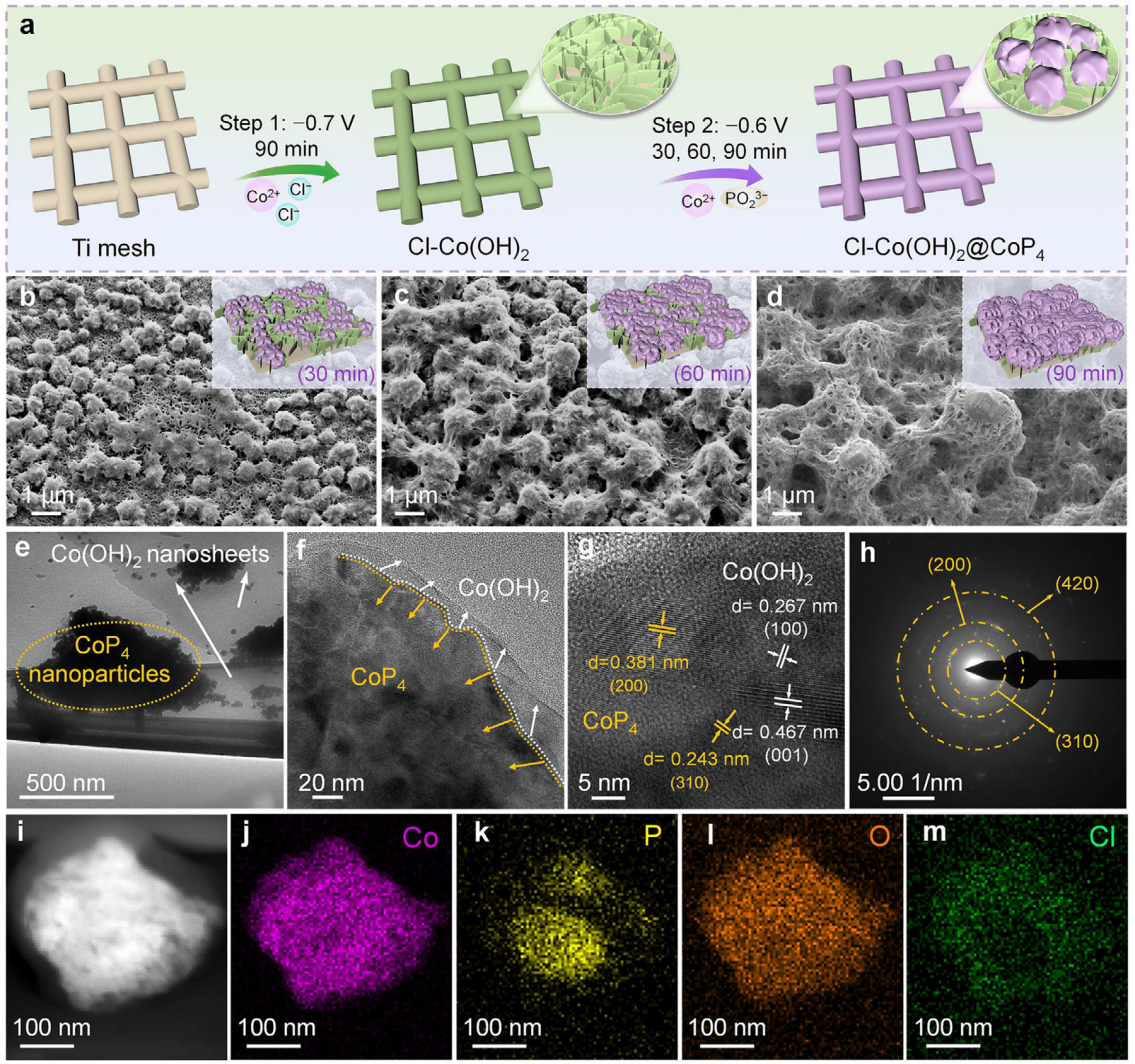
(a) Schematic diagram of the synthesis process of Cl‐Co(OH)_2_@CoP_4_. (b–d) SEM images of Cl‐Co(OH)_2_@CoP_4_‐30, 60, and 90, respectively. (e) TEM and (f,g) HRTEM images of Cl‐Co(OH)_2_@CoP_4_‐60. (h) SAED pattern and (i‐m) elemental mapping images of Cl‐Co(OH)_2_@CoP_4_‐60.

The detailed microstructure of the Cl‐Co(OH)_2_@CoP_4_ heterostructure was further characterized by TEM. As shown in Figure 1e, CoP_4_ nanoparticles are uniformly anchored on the nanosheet surface, forming a tightly coupled heterogeneous interface. The corresponding high‐resolution TEM images (Figure 1f,g) reveal clear lattice fringes with interplanar spacings of 0.467 and 0.267 nm assigned to (001) and (100) crystal planes of Cl‐Co(OH)_2_, and 0.381 and 0.243 nm corresponding to (200) and (310) crystal planes of CoP_4_, respectively, confirming the successful construction of the heterostructure. In addition, the selected area electron diffraction pattern exhibits weak diffraction rings indexed to the (310), (200), and (420) crystal planes of CoP_4_ (Figure 1h), which can be attributed to the relatively low crystallinity of CoP_4_ formed during electrodeposition. The scanning TEM images and corresponding elemental mapping results further demonstrate the uniform spatial distribution of Co (69.63 at. %), P (7.94 at. %), O (20.39 at. %), and Cl (0.22 at. %) elements across the nanosheet arrays (Figure 1i–m and Figure S5), confirming that Cl is successfully incorporated into the Co(OH)_2_ lattice while phosphorus species are mainly concentrated in the outer CoP_4_ nanoparticles. Such a hierarchical heterostructure with lattice‐level Cl incorporation and surface‐confined phosphorus‐rich nanoparticles provides an ideal platform for constructing an interfacial electronic coupling effect between the inner active layer and outer protective layer [28, 29], which is essential for simultaneously regulating catalytic activity and corrosion resistance during seawater electrolysis.

Figure S6 further compares the morphology of Co(OH)_2_ nanosheets modified with different halogen dopants. In contrast to the relatively flat and well‐defined nanosheet arrays observed for Cl‐Co(OH)_2_, the F‐, Br‐, and I‐ modified Co(OH)_2_ nanosheets exhibit noticeably more compact and aggregated structures, suggesting that different halogen species play distinct roles in regulating nanosheet growth during electrodeposition. Energy‐dispersive spectroscopy (EDS) analysis confirms the successful incorporation of halogen elements into the hydroxide framework, with atomic contents of 9.95, 10.26, 10.41, and 11.82 at. % for F, Cl, Br, and I, respectively. Moreover, the size and distribution of CoP_4_ nanoparticles grown on X‐Co(OH)_2_ (X = F, Cl, Br, I) surfaces show clear differences (Figure S7). Compared with the other halogen‐modified counterparts, Cl‐Co(OH)_2_ enables the formation of more uniformly distributed CoP_4_ nanoparticles and a more compact heterointerfacial structure, indicating that lattice Cl incorporation is particularly favorable for constructing well‐defined heterostructures.

Next, the crystalline structures of the samples were analyzed by x‐ray diffraction (XRD). As shown in Figure 2a, the electrodeposited cobalt hydroxide exhibits characteristic diffraction peaks at 32.6°, 33.4°, 37.9°, 58.3°, and 60.4°, corresponding to Co(OH)_2_ (JCPDS no. 02–0925). After surface modification, additional diffraction peaks located at 16.4°, 36.9°, 47.3°, and 53.2° are assigned to the (100), (310), (400), and (420) planes of CoP_4_ (JCPDS no. 20–0336), respectively, confirming the successful formation of CoP_4_ on the nanosheet surface. Notably, the characteristic diffraction peaks of Co(OH)_2_ become less distinguishable after CoP_4_ deposition, which can be attributed to the uniform surface coverage of the nanosheets by CoP_4_ nanoparticles, further indicating the formation of a conformal heterostructure. To further investigate the surface chemical states and electronic structures of the samples, x‐ray photoelectron spectroscopy (XPS) measurements were performed. The survey spectra confirm the presence of Co, O, P, and Cl elements in Cl‐Co(OH)_2_@CoP_4_ (Figure S8), consistent with the elemental mapping results. In the high‐resolution Co 2p spectrum, compared with pristine Co(OH)_2_, the binding energies of Co^2+^ and Co^3+^ in Cl‐Co(OH)_2_ shift toward lower values (Figure 2b and Table S1), which is attributed to the increased electron cloud density around the Co atoms induced by Cl incorporation [30]. In Cl‐Co(OH)_2_@CoP_4_, a characteristic Co–P peak appears at 777.92 eV, while the peak positions of Co^2+^ (782.68 eV) and Co^3+^ (780.73 eV) are close to those of Co(OH)_2_. The increased Co^2+^/Co^3+^ ratio reflects a reduced degree of surface oxidation by air. The high‐resolution O 1s spectrum (Figure 2c) can be deconvoluted into three components corresponding to adsorbed oxygen species, metal–hydroxyl bonds, and metal–oxygen bonds. The increased contribution of metal–oxygen species in Cl‐Co(OH)_2_@CoP_4_ suggests partial oxidation of the phosphorus‐rich surface layer. The Cl 2p spectra (Figure 2d) further confirm that the Cl species are mainly present in lattice form rather than physically adsorbed chloride ions, indicating successful lattice incorporation of Cl into the Co(OH)_2_ structure [31, 32]. Meanwhile, the high‐resolution P 2p spectrum (Figure 2e) shows characteristic peaks corresponding to both P–O and metal–P bonds, demonstrating partial oxidation of surface CoP_4_ species and the coexistence of phosphorus‐rich protective components.

**FIGURE 2:**
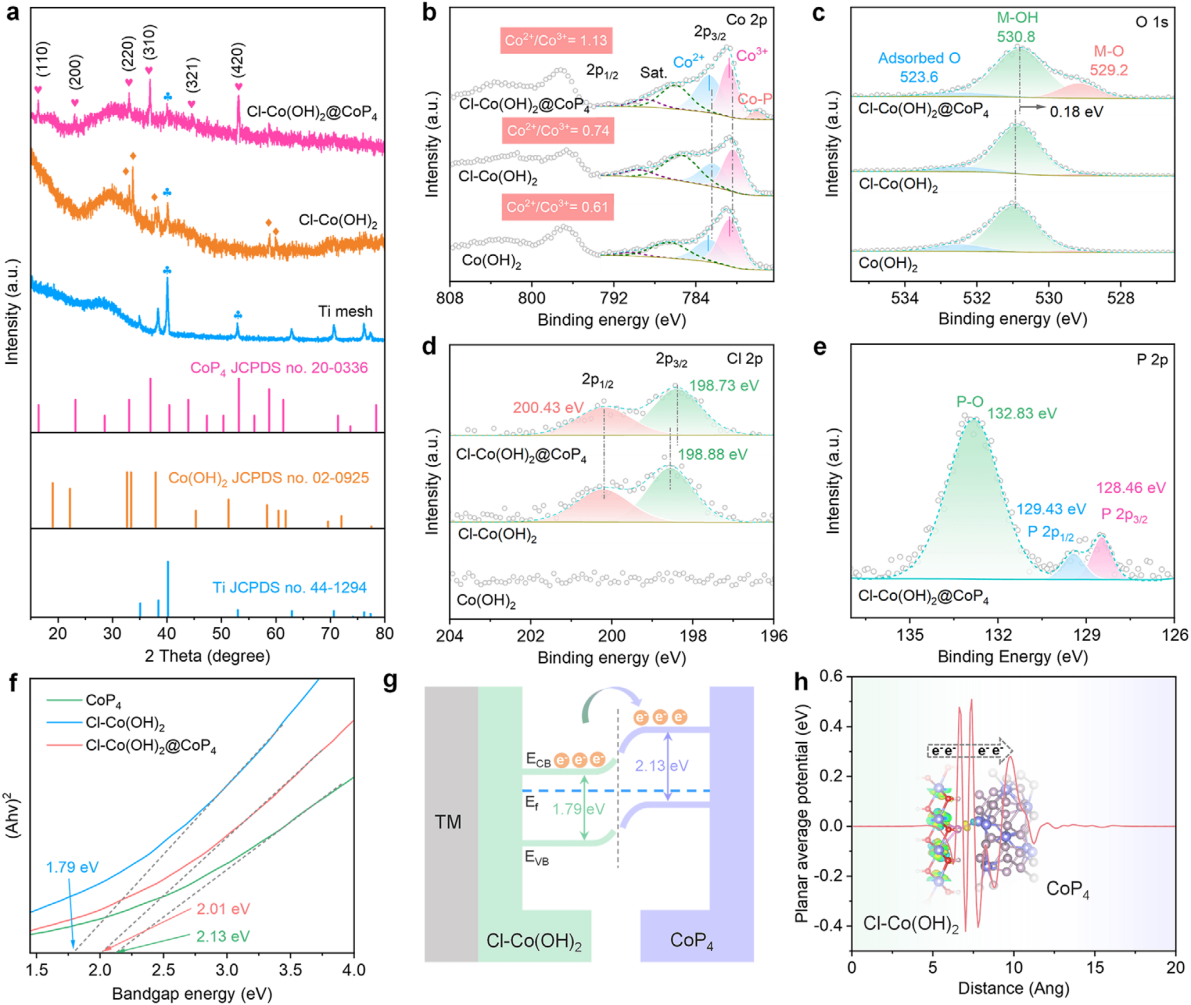
(a) XRD patterns of Cl‐Co(OH)_2_ and Cl‐Co(OH)_2_@CoP_4_. High‐resolution XPS spectra for (b) Co 2p, (c) O 1s, (d) Cl 2p, (e) P 2p. (f) UV/Vis DRS spectra of CoP_4_, Cl‐Co(OH)_2_ and Cl‐Co(OH)_2_@CoP_4_. (g) Energy band diagram of Cl‐Co(OH)_2_ and CoP_4_ in the heterostructure. (h) Planer average potential diagram of the interface between Cl‐Co(OH)_2_ and CoP_4_.

These XPS results confirm that lattice Cl‐modified Co(OH)_2_ and the phosphorus‐rich surface CoP_4_ component form a coupled inner–outer heterostructure with strong interfacial electronic interaction. Such heterointerface formation induces charge redistribution across the interface to reach Fermi‐level equilibration between the two components [33, 34]. To further clarify the direction of interfacial electron transfer, ultraviolet/visible diffuse reflectance spectroscopy and work‐function analysis were subsequently employed [35]. As shown in Figure 2f, Cl‐Co(OH)_2_@CoP_4_ exhibits a narrower bandgap (2.01 eV) compared with CoP_4_ (2.13 eV), indicating enhanced electronic interaction between CoP_4_ nanoparticles and Cl‐Co(OH)_2_ substrate. As shown in Figure S9, density functional theory (DFT) calculations further reveal that the work function of Cl‐Co(OH)_2_ (3.29 eV) is lower than that of CoP_4_ (4.49 eV), indicating that electrons tend to transfer from Cl‐Co(OH)_2_ to CoP_4_ across the heterointerface (Figure 2g). The plane‐averaged potential distribution further confirms electron depletion in Cl‐Co(OH)_2_ and electron accumulation in CoP_4_ (Figure 2h), indicating the presence of electron transfer at the heterointerface. This interfacial charge redistribution is expected to optimize the adsorption behavior of oxygen‐containing intermediates and promote selective OH adsorption while suppressing competitive chloride adsorption during seawater electrolysis [36, 37].

### 2.2 Catalytic Activity and Surface Reconstruction Behavior

The influence of lattice Cl incorporation on catalytic activity was first evaluated in alkaline electrolyte. Compared with pristine Co(OH)_2_, Cl‐Co(OH)_2_ exhibits markedly enhanced OER activity (Figure S10), indicating that lattice Cl incorporation effectively modulates the electronic structure of catalytically active Co centers. Optimization of the Cl doping level further reveals that the sample prepared with 0.1 M KCl delivers the highest activity (Figure S11). Notably, among four halogen‐modified counterparts, Cl‐Co(OH)_2_ still exhibits the best catalytic performance (Figure S12), confirming that lattice Cl incorporation provides the most favorable electronic regulation for activating Co sites toward oxygen evolution. After surface decoration with CoP_4_ nanoparticles, the catalytic activity is further significantly enhanced. To intuitively compare the catalytic performance of different materials, a two‐dimensional OER activity map under alkaline conditions is plotted using linear sweep voltammetry curves (Figure 3a), revealing the OER activity sequence as follows: Cl‐Co(OH)_2_@CoP_4_‐60> Cl‐Co(OH)_2_@CoP_4_‐30> Cl‐Co(OH)_2_@CoP_4_‐90> IrO_2_. Among them, Cl‐Co(OH)_2_@CoP_4_‐60 requires overpotentials of only 358 and 405 mV to reach current densities of 500 and 1000 mA cm^−2^, respectively, together with a Tafel slope of 100.6 mV dec^−1^ (Figure S13), demonstrating accelerated OER kinetics after heterostructure construction [38, 39]. Its performance is competitive with recently reported state‐of‐the‐art OER catalysts (Figure S14 and Table S2). Electrochemical impedance spectroscopy (EIS) further shows that Cl‐Co(OH)_2_@CoP_4_‐60 possesses the smallest charge‐transfer resistance among all samples (Figure S15), indicating facilitated interfacial electron transport enabled by heterointerface formation. Meanwhile, double‐layer capacitance measurements reveal the largest electrochemically active surface area for Cl‐Co(OH)_2_@CoP_4_‐60 (Figure S16 and S17), suggesting increased exposure of accessible catalytic sites. The markedly enhanced catalytic performance therefore originates from the synergistic coupling between lattice Cl‐induced electronic regulation and interfacial electronic interaction with CoP_4_, which collectively optimizes the adsorption energetics of oxygen‐containing intermediates and accelerates the overall OER process.

**FIGURE 3:**
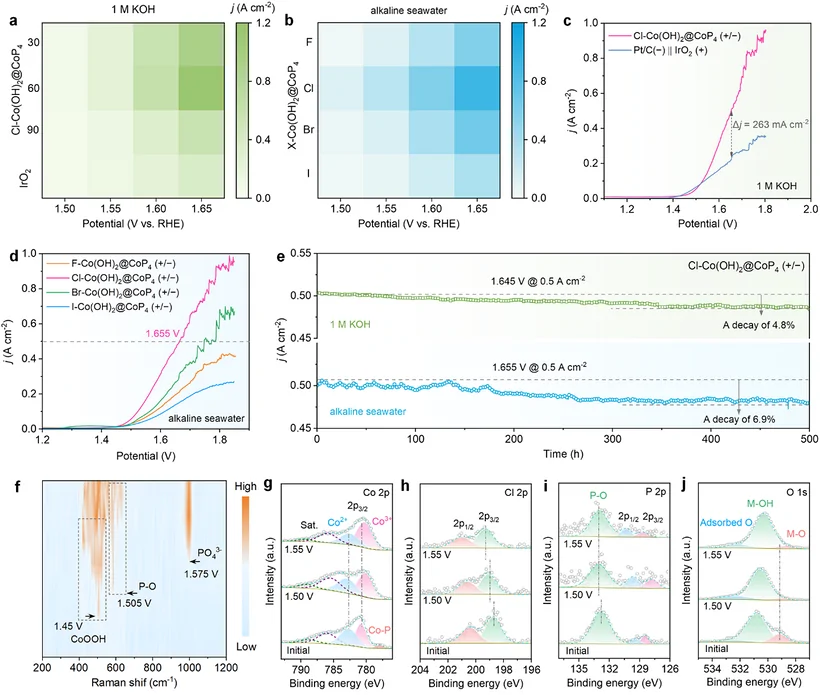
Potential‐dependent partial current densities of *j*_OER_ for (a) Cl‐Co(OH)_2_@CoP_4_‐30, 60, 90, and (b) X‐Co(OH)_2_@CoP_4_ (X = F, Cl, Br, I). (c) OWS performance based on Cl‐Co(OH)_2_@CoP_4_ (+/−) and Pt/C (−) || IrO_2_ (+) couples. (d) OWS performance of X‐Co(OH)_2_@CoP_4_ couples (X = F, Cl, Br, I) in alkaline seawater. (e) Long‐term stability test for overall water splitting in 1.0 m KOH an alkaline seawater. (f) The in situ Raman spectroscopy in alkaline seawater solution at the surface of Cl‐Co(OH)_2_@CoP_4_. High‐resolution XPS spectroscopy of (g) Co 2p, (h) Cl 2p, (i) P 2p, and (j) O 1s under different voltage.

To further evaluate the practical applicability of the halogen‐modified catalysts under realistic seawater electrolysis conditions, the OER performance of Co(OH)_2_ and X‐Co(OH)_2_ (X = F, Cl, Br, I) was first investigated in alkaline seawater electrolyte. As shown in Figures S18 and S19, Cl‐Co(OH)_2_ still exhibits the highest catalytic activity among all halogen‐modified counterparts, indicating that lattice Cl incorporation remains effective in regulating catalytic selectivity and activity even in chloride‐rich environments. The competition between OER and ClER was subsequently examined by systematically varying the NaCl concentration in alkaline electrolyte. When the NaCl concentration is lower than 0.4 m, the overpotential gradually increases (Figure S20), suggesting that chloride ions partially occupy surface active sites without participating in the reaction. At higher NaCl concentrations, the overpotential begins to decrease and subsequently reaches a plateau, indicating effective suppression of the competing ClER side reaction. These observations confirm that the synergistic regulation between lattice Cl incorporation and surface CoP_4_ modification effectively preserves OER activity under saline conditions by mitigating chloride‐induced activity loss. Figure 3b further compares the OER performance of X‐Co(OH)_2_@CoP_4_ (X = F, Cl, Br, I) in alkaline seawater electrolyte. Among them, Cl‐Co(OH)_2_@CoP_4_ exhibits the best catalytic performance, requiring overpotentials of only 262 and 358 mV to achieve current densities of 100 and 500 mA cm^−2^, respectively (Figure S21), demonstrating that the optimized heterostructure maintains fast reaction kinetics even in chloride‐containing electrolyte. Electrochemical surface area analysis reveals that Cl‐Co(OH)_2_@CoP_4_ possesses the largest double‐layer capacitance and electrochemically active surface area among all samples (Figures S22 and S23), indicating increased exposure of accessible catalytic sites. Meanwhile, EIS measurements show that Cl‐Co(OH)_2_@CoP_4_ exhibits the smallest charge‐transfer resistance in alkaline seawater electrolyte (Figure S24), confirming accelerated interfacial electron‐transfer kinetics enabled by heterointerface formation. In addition, multicycle and current‐step measurements demonstrate excellent operational stability of Cl‐Co(OH)_2_@CoP_4_ under alkaline seawater conditions (Figure S25). These results demonstrate that lattice Cl incorporation not only regulates the electronic structure of catalytic centers but also cooperatively enhances interfacial charge‐transfer capability through coupling with CoP_4_, thereby enabling efficient and selective OER catalysis under high‐salinity conditions. Using a similar surface heterostructure construction strategy, two additional catalysts, Cl‐Co(OH)_2_@Co_3_S_4_ and Cl‐Co(OH)_2_@CoSe_2_, were synthesized for comparison. As shown in Figures S26 and S30, the formation of these surface heterostructures leads to distinct interfacial morphologies on the Cl‐Co(OH)_2_ nanosheets, while the structural integrity of the nanosheet framework remains well preserved. Electrochemical measurements reveal that these heterostructured catalysts exhibit comparable OER activity in alkaline electrolyte (Figure S31). In contrast, Cl‐Co(OH)_2_@CoP_4_ delivers markedly superior catalytic performance during alkaline seawater electrolysis (Figure S32), which can be attributed to the more effective interfacial protection effect and optimized charge‐transfer behavior introduced by the phosphorus‐rich CoP_4_ surface layer.

Beyond oxygen evolution catalysis in both alkaline and seawater electrolytes, the hydrogen evolution reaction (HER) performance of the as‐prepared catalysts was further evaluated to assess their bifunctional electrocatalytic capability toward overall water splitting. Among all samples, Cl‐Co(OH)_2_@CoP_4_‐60 exhibits superior HER activity compared with commercial Pt/C catalysts at high current densities (*j* > 425 mA cm^−2^) in 1.0 m KOH (Figure S33), together with a low Tafel slope of 70.8 mV dec^−1^, indicating favorable HER kinetics under alkaline conditions [40, 41]. In particular, Cl‐Co(OH)_2_@CoP_4_‐60 requires overpotentials of only 123, 181, and 221 mV to achieve current densities of 100, 500, and 1000 mA cm^−2^, respectively, demonstrating its capability and competitive advantage for efficient hydrogen production at industrially relevant current densities (Table S3). More importantly, Cl‐Co(OH)_2_@CoP_4_‐60 maintains the best catalytic activity among all samples in alkaline seawater electrolyte. As shown in Figure S34, an overpotential of only 218 mV is required to deliver 500 mA cm^−2^ for HER, together with the lowest Tafel slope, demonstrating accelerated hydrogen evolution kinetics under chloride‐containing conditions. As shown in Figures S35 and S36, Cl‐Co(OH)_2_@CoP_4_‐60 exhibits the smallest charge transfer resistance in alkaline seawater, while negligible activity decay is observed after 5000 CV cycles and 600 h of continuous operation, highlighting the excellent durability of the catalyst under practical operating conditions. The combination of high catalytic activity toward both OER and HER therefore enables the construction of efficient symmetric electrolyzers based on Cl‐Co(OH)_2_@CoP_4_, providing a promising platform for practical seawater electrolysis.

Encouraged by the outstanding bifunctional catalytic performance, Cl‐Co(OH)_2_@CoP_4_‐60 was further employed as both the anode and cathode catalyst for overall water splitting (OWS). As shown in Figure 3c, the symmetric electrolyzer requires only 1.645 V to deliver a current density of 500 mA cm^−2^, outperforming the benchmark Pt/C–IrO_2_ electrode pair under identical conditions. The overall seawater electrolysis performance of X‐Co(OH)_2_@CoP_4_ (X = F, Cl, Br, I) was further evaluated in alkaline seawater electrolyte (Figure 3d). Among them, Cl‐Co(OH)_2_@CoP_4_ achieves 500 mA cm^−2^ at a low cell voltage of only 1.655 V, surpassing the corresponding F‐, Br‐, and I‐modified heterostructures. The measured H_2_/O_2_ production ratio was close to the theoretical value of 2:1 (Figure S37), with Faradaic efficiencies (FE) of 97.7% and 90.6% for the HER and OER, respectively. The slightly lower OER Faradaic efficiency may be attributed to the further oxidation of the catalyst during operation, resulting in partial charge consumption associated with catalyst oxidation rather than oxygen evolution. Comparison with previously reported catalysts further highlights the superior bifunctional catalytic performance of Cl‐Co(OH)_2_@CoP_4_ for alkaline seawater electrolysis (Figure S38 and Table S4). Moreover, the Cl‐Co(OH)_2_@CoP_4_ electrolyzer exhibits excellent long‐term durability in both 1.0 M KOH and alkaline seawater electrolytes. Only minor voltage increases of 4.8% and 6.9% are observed after continuous operation for over 500 h at a current density of 500 mA cm^−2^ (Figure 3e), demonstrating remarkable operational stability under industrially relevant conditions. These results collectively demonstrate that the cooperative regulation arising from lattice Cl incorporation and surface CoP_4_ heterointerface formation simultaneously enhances intrinsic catalytic activity and structural robustness, thereby enabling efficient and durable high‐current‐density seawater electrolysis.

To clarify the structural evolution of the catalyst during electrolysis, the post‐reaction electrodes were first characterized by XRD after long‐term stability testing [42, 43]. The diffraction results reveal that the surface CoP_4_ phase is partially transformed into cobalt phosphate species (Co_3_(PO_4_)_2_, JCPDS No. 70–2082) (Figure S39), confirming the occurrence of in situ surface reconstruction during electrolysis. Notably, distinct morphological differences are observed after anodic operation in different electrolytes. Following alkaline OER testing, the surface particles evolve into a loose and porous structure (Figure S40a,b), whereas larger particles with partially blocked pore structures are observed after operation in alkaline seawater electrolyte (Figure S40c,d), suggesting electrolyte‐dependent reconstruction behavior. High‐resolution TEM images further display clear lattice fringes corresponding to the (−101), (−111), (031), and (210) planes of Co_3_(PO_4_)_2_ (Figure S41), providing direct evidence for the formation of phosphate species after electrolysis. Consistently, high‐resolution Co 2p and P 2p XPS spectra show the disappearance of the initial Co–P signal after reaction (Figure S42), further confirming the transformation of surface CoP_4_ into phosphate species. Similar structural and surface chemical evolution is also observed for the cathodic electrodes after long‐term operation (Figures S43 and S44).

In situ Raman spectroscopy was conducted at different applied potentials to further probe the dynamic formation of the phosphate passivation layer [44]. Cl‐Co(OH)_2_ exhibits characteristic Co–O(H) and Co–O vibrational modes, whereas Cl‐Co(OH)_2_@CoP_4_ shows a weakened and broadened Co–O signal together with a distinct Co–P vibration (Figure S45), indicating strong interfacial coupling between CoP_4_ nanoparticles and the hydroxide framework [45]. As shown in Figure 3f, the emergence of the Co–O vibration at 1.45 V indicates the formation of CoOOH active species during anodic polarization. With further increase of the applied potential, characteristic P–O and PO_4_^3−^ vibrational features gradually appear [25, 46], confirming that the surface CoP_4_ layer undergoes in situ transformation into phosphate species under seawater electrolysis conditions. The dynamically generated phosphate species thus form a reconstructed surface layer that selectively suppresses chloride adsorption while maintaining efficient OH^−^ transport toward catalytic active sites, thereby simultaneously enhancing catalytic activity and corrosion resistance during seawater electrolysis. Corresponding XPS analysis further reveals potential‐dependent electronic structure modulation during anodic polarization [47, 48]. The Co 2p_3/2_ peak shifts toward lower binding energy, while the Cl 2p_3/2_ and P–O signals shift toward higher binding energies (Figure 3g–i), indicating increased electron density redistribution around Co centers induced by cooperative Cl and phosphate coordination. It should be noted that a small amount of pre‐existing surface P–O species originates from unavoidable air oxidation, while anodic polarization further promotes the sustained formation and enrichment of phosphate species, ultimately leading to the formation of a phosphate‐rich protective surface layer. Meanwhile, the metal–oxygen component in the O 1s spectrum decreases in intensity and shifts toward lower binding energy (Figure 3j), further supporting the formation of a dynamically reconstructed phosphate‐rich surface environment. This reconstructed phosphate layer therefore acts as an effective chloride‐resistant interfacial barrier, which stabilizes catalytic active sites and enhances corrosion resistance under alkaline seawater electrolysis conditions.

### 2.3 Theoretical Calculations

Based on the above experimental results, to further elucidate the origin of the enhanced catalytic activity and corrosion resistance of Cl‐Co(OH)_2_@CoP_4_, DFT calculations are performed to investigate the effects of lattice Cl incorporation and heterostructure formation on reaction energetics and adsorption behavior. The optimized structural models of CoP_4_, Co(OH)_2_@CoP_4_, and Cl‐Co(OH)_2_@CoP_4_ are shown in Figure S46. The Gibbs free energy diagrams for the HER process are first calculated to evaluate hydrogen adsorption behavior on different catalysts [49, 50]. As shown in Figure S47, Cl‐Co(OH)_2_@CoP_4_ exhibits a nearly thermoneutral hydrogen adsorption free energy (|Δ*G*_H*_| = 0.08 eV), which is significantly lower than those of CoP_4_ and Co(OH)_2_@CoP_4_ and even comparable to Pt, indicating that lattice Cl incorporation and heterointerface formation synergistically optimize hydrogen adsorption/desorption behavior.

To clarify the origin of the enhanced corrosion resistance, a dual‐protection mechanism involving lattice Cl shielding and surface phosphate reconstruction is proposed, as illustrated in Figure 4a. On pristine Co(OH)_2_ surfaces, catalytic active sites are easily occupied by chloride ions, resulting in competitive reactions and the formation of unstable MCl_x_^−^ species. In contrast, lattice Cl incorporation within Cl‐Co(OH)_2_ forms a shielding environment around catalytic centers, effectively suppressing chloride‐induced corrosion reactions. Following the introduction of the CoP_4_ surface layer, in situ anodic oxidation generates phosphate species, which subsequently construct a phosphate‐rich protective interfacial layer, giving rise to a coupled inner–outer corrosion‐resistant architecture. This cooperative inner‐shielding/outer‐protection configuration effectively reduces the accessibility of chloride species to the catalytic active sites while preserving favorable OH^−^ adsorption and oxygen evolution kinetics during seawater electrolysis.

**FIGURE 4:**
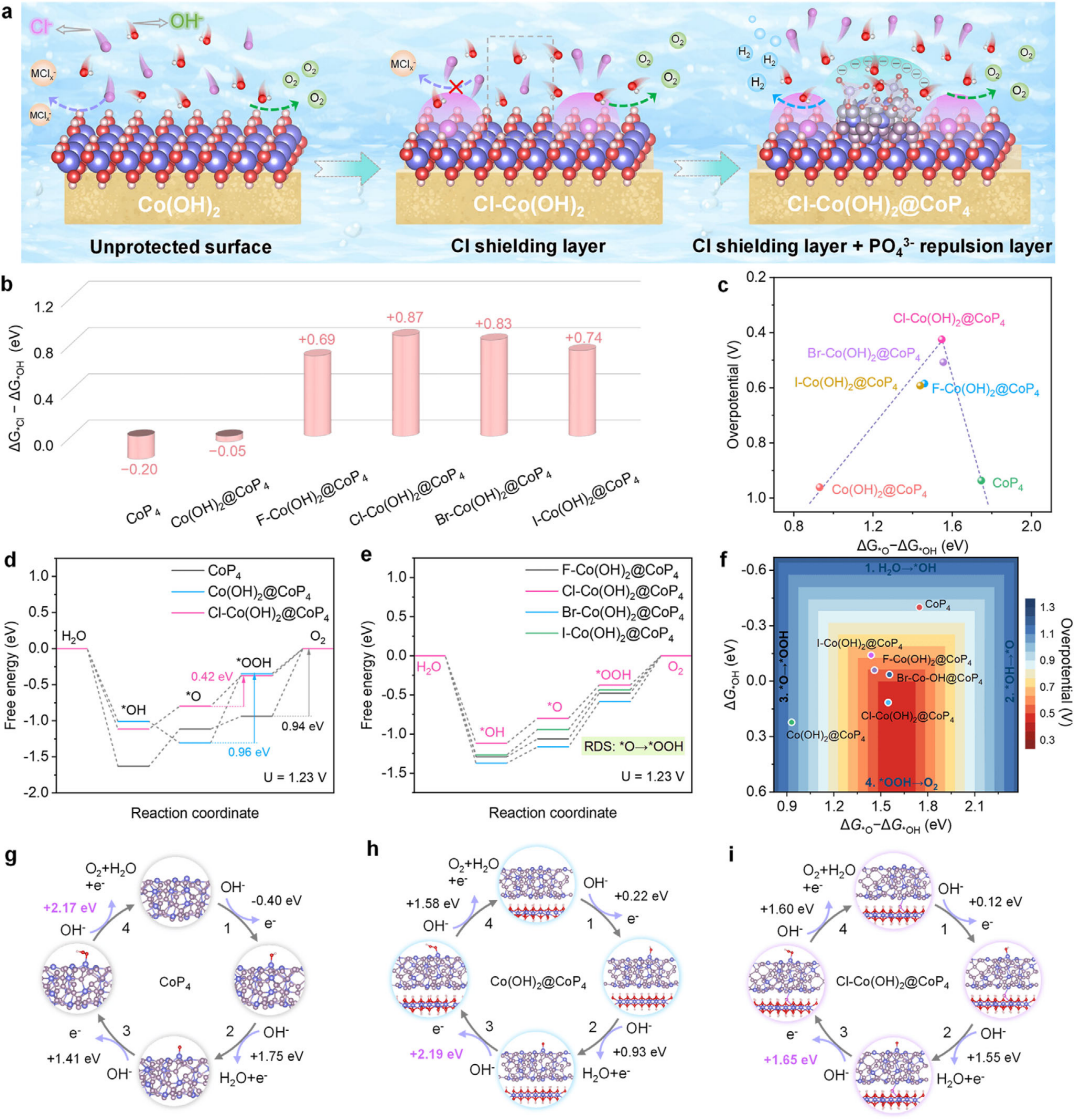
(a) Schematic diagram of a seawater electrolysis mechanism: dual corrosion‐resistant mechanism involving chloride ion doping and surface phosphates. DFT calculation results for different materials: (b) The adsorption energy of OH^−^ and Cl^−^. (c) Volcano plot of overpotential vs. reaction free‐energy change. (d,e) Gibbs free energy diagram for OER. (f) 2D OER activity map of theoretical overpotentials regarding different dopants. Reaction pathway diagram for the four steps of the OER on (g) CoP_4_, (h) Co(OH)_2_@CoP_4_ and (i) Cl‐Co(OH)_2_@CoP_4_.

As shown in Figure 4b, the adsorption energy difference between Cl^−^ and OH^−^ species on different catalyst models was calculated to elucidate the adsorption preference of reactive intermediates at the catalytic interface. The negative adsorption energy differences observed for CoP_4_ and Co(OH)_2_@CoP_4_ indicate a stronger affinity toward Cl^−^ adsorption. In contrast, after halide‐ion (F, Cl, Br, I) incorporation, the adsorption energy difference becomes significantly more positive, suggesting that halide modification effectively tunes the catalyst surface to preferentially adsorb OH^−^ while suppressing Cl^−^ adsorption. These results further demonstrate that halide ions can optimize the interfacial adsorption behavior, thereby enhancing OER selectivity over chlorine‐related side reactions. Projected density of states analysis further reveals that, after lattice Cl incorporation, the Co d orbital electronic states in Cl‐Co(OH)_2_@CoP_4_ shift toward the Fermi level, accompanied by increased electronic states near the Fermi level (Figure S48). This suggests enhanced electronic conductivity and improved interfacial charge transfer capability. Such electronic structure modulation strengthens the electronic interaction between catalytic active sites and reaction intermediates, thereby optimizing the adsorption behavior of oxygen‐containing intermediates and accelerating OER kinetics. Meanwhile, the regulated electronic structure of Co active sites also helps weaken competitive Cl^−^ adsorption, favoring selective OH^−^ adsorption during seawater electrolysis. The catalytic activity differences among halogen‐modified catalysts were further analyzed using a volcano relationship between overpotential and reaction free‐energy descriptors (Figure 4c). Cl‐Co(OH)_2_@CoP_4_ is located near the top of the volcano plot, indicating its optimized intermediate adsorption configuration and lowest theoretical overpotential among all models.

To gain mechanistic insights into the OER process within an adsorbate evolution mechanism (AEM)‐based theoretical framework, the Gibbs free energy changes of oxygen‐containing intermediates were calculated for different catalyst models, allowing the potential‐limiting step (PLS) to be identified [51, 52]. As shown in Figure S49, the PLS of Cl‐Co(OH)_2_@CoP_4_ corresponds to the *O → *OOH transformation with a free‐energy change of 1.65 eV under U = 0 V, which is significantly lower than that of Co(OH)_2_@CoP_4_ (1.92 eV). Even under the theoretical operating potential (U = 2.19 V), Cl‐Co(OH)_2_@CoP_4_ still exhibits the lowest reaction free‐energy of 0.42 eV (Figure 4d), confirming the optimized reaction pathway after lattice Cl incorporation. Furthermore, comparative analysis of halogen‐doped catalysts demonstrates that all X‐Co(OH)_2_@CoP_4_ (X = F, Cl, Br, I) catalysts share the same PLS but exhibit different free‐energy changes (Figure 4e and Figure S50), among which Cl‐Co(OH)_2_@CoP_4_ possesses the lowest free‐energy change. A two‐dimensional activity map based on theoretical overpotentials further confirms that Cl‐Co(OH)_2_@CoP_4_ exhibits the most favorable catalytic activity among all investigated models (Figure 4f). Reaction pathway diagrams for CoP_4_, Co(OH)_2_@CoP_4_, and Cl‐Co(OH)_2_@CoP_4_ further illustrate that the optimized electronic structure induced by lattice Cl incorporation and heterointerface construction significantly reduces the free‐energy changes of key reaction steps (Figure 4g–i). As shown in Figure 4i and Figures S51–S53, the four‐electron reaction pathway diagrams of X‐Co(OH)_2_@CoP_4_ (X = F, Cl, Br, I) more clearly reveal that the PLS remains unchanged, while the corresponding free‐energy changes gradually increase across the halogen series, indicating that halogen incorporation plays a critical role in regulating the reaction energetics. Overall, the theoretical calculations demonstrate that the synergistic interaction between lattice Cl incorporation and dynamically reconstructed phosphate species simultaneously optimizes intermediate adsorption behavior and suppresses chloride poisoning effects, thereby enabling highly efficient and stable seawater electrolysis.

### 2.4 Performance of Flow‐Through Symmetric Electrolyzer

The practical seawater electrolysis performance of Cl‐Co(OH)_2_@CoP_4_ was further evaluated using a flow‐through symmetric electrolyzer configuration. Cl‐Co(OH)_2_@CoP_4_ exhibits enhanced OER activity and faster reaction kinetics compared with Co(OH)_2_@CoP_4_ in both alkaline electrolyte and alkaline seawater conditions (Figure 5a,b), confirming the effectiveness of lattice Cl regulation under chloride‐containing environments. Tafel corrosion analysis further reveals a more positive corrosion potential and lower corrosion current density for Cl‐Co(OH)_2_@CoP_4_ (Figure 5c), indicating improved resistance toward chloride‐induced corrosion. Furthermore, the generation of hypochlorite (ClO^−^) was quantitatively analyzed using a UV–vis colorimetric method (Figure S54a,b). The results show that the OER Faradaic efficiency of Cl‐Co(OH)_2_@CoP_4_ remained above 95% over the investigated polarization potentials, while the Faradaic efficiency for the chlorine evolution reaction (ClER) was maintained below 4% (Figure S54c,d). As shown in Figure S54e, Cl‐Co(OH)_2_@CoP_4_ produced a substantially lower hypochlorite concentration than Co(OH)_2_@CoP_4_ during seawater electrolysis, indicating that competitive chlorine evolution was effectively suppressed and OER selectivity was significantly enhanced. More importantly, long‐term seawater electrolysis demonstrated that Cl‐Co(OH)_2_@CoP_4_ maintained stable oxygen evolution activity with an OER Faradaic efficiency of approximately 96% throughout the durability test (Figure S54f). These results demonstrate that the synergistic effect of lattice chloride shielding and the dynamically reconstructed phosphate layer not only enhances corrosion resistance but also regulates reaction selectivity toward oxygen evolution during seawater electrolysis.

**FIGURE 5:**
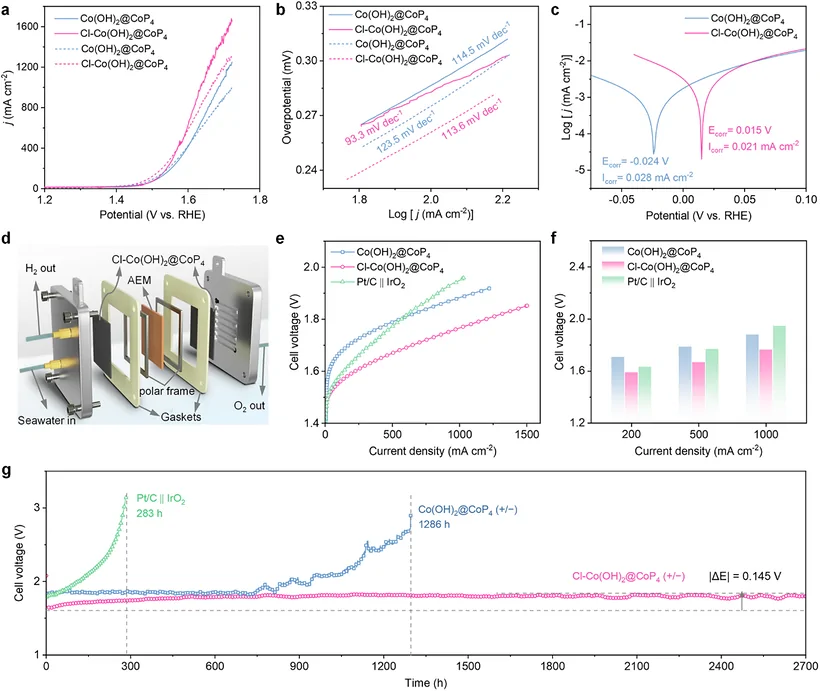
(a) The OER polarization curves and (b) Tafel plots of Co(OH)_2_@CoP_4_ and Cl‐Co(OH)_2_@CoP_4_ in 1.0 m KOH (full line) and alkaline seawater (dotted line). (c) Tafel plots of Co(OH)_2_@CoP_4_ and Cl‐Co(OH)_2_@CoP_4_ in alkaline seawater. (d) Schematic diagram of flow‐through electrolyser. (e) Polarization curves of alkaline seawater electrolyser (ASWE) using Co(OH)_2_@CoP_4_ || Co(OH)_2_@CoP_4_, Cl‐Co(OH)_2_@CoP_4_ || Cl‐Co(OH)_2_@CoP_4_, Pt/C || IrO_2_. (f) Corresponding voltage at different current densities of Cl‐Co(OH)_2_@CoP_4_ and control samples. (g) Durability test at a constant current density of 500 mA cm^−2^ in the alkaline seawater electrolyser.

A symmetric alkaline seawater electrolyzer assembled using Cl‐Co(OH)_2_@CoP_4_ as both electrodes delivers a current density of 500 mA cm^−2^ at 1.67 V, outperforming Co(OH)_2_@CoP_4_ and commercial noble‐metal‐based electrode systems (Figure 5d–f). The device performance is also comparable to or better than recently reported state‐of‐the‐art bifunctional catalysts (Figure S55 and Table S5). Notably, operation at 500 mA cm^−2^ falls within the industrially relevant current‐density range for practical seawater electrolysis. The symmetric electrolyzer of Cl‐Co(OH)_2_@CoP_4_ maintains stable operation for over 2700 h at 500 mA cm^−2^ (Figure 5g), far exceeding the durability of Pt/C || IrO_2_ (283 h) and Co(OH)_2_@CoP_4_ (1286 h) under identical conditions. Such sustained high‐current‐density operation verifies that the cooperative protection mechanism originating from lattice Cl shielding and in situ generated phosphate armor effectively preserves catalytic active sites and suppresses chloride‐induced degradation during prolonged seawater electrolysis.

## 3 Conclusion

In summary, we report a Cl‐Co(OH)_2_@CoP_4_ heterostructured electrocatalyst featuring lattice chloride regulation coupled with dynamically reconstructed phosphate protection for efficient and selective hydrogen production by alkaline seawater electrolysis. Lattice Cl incorporation modulates the electronic structure of Co active sites and suppresses chloride‐induced corrosion, while the surface CoP_4_ layer undergoes in situ transformation into phosphate species under anodic polarization, forming a protective outer layer. The cooperative interaction between lattice chloride shielding and the dynamically generated phosphate layer establishes a dual‐protection mechanism that simultaneously promotes selective OH^−^ adsorption and suppresses competing chlorine evolution reactions during seawater electrolysis. Density functional theory calculations further reveal a consistent halogen‐dependent regulation trend, in which halogen incorporation weakens chloride adsorption while lowering the reaction free‐energy changes for both hydrogen and oxygen evolution, thereby confirming the critical role of lattice halogen regulation in optimizing catalytic selectivity and reaction kinetics. Benefiting from this cooperative regulation strategy, a symmetric Cl‐Co(OH)_2_@CoP_4_ electrolyzer delivers a current density of 500 mA cm^−2^ at only 1.67 V and maintains stable operation for over 2700 h in alkaline seawater. More broadly, this work establishes a dual‐protection electrocatalytic strategy that integrates intrinsic electronic regulation with dynamic surface reconstruction, providing new design principles for developing corrosion‐resistant electrocatalysts toward practical hydrogen production by seawater electrolysis.

## Author Contributions

**Yatao Yan**: data curation, conceptualization, writing – original draft, validation, investigation. **Jifang Chen**: software, conceptualization. **Ke Zhou**: visualization, formal analysis. **Xing Chen**: software, validation, methodology. **Lubin Ni**: methodology, investigation, resources. **Shuo Yang**: investigation, data curation. **Ming Chen**: writing – review and editing, project administration, resources, supervision, funding acquisition. **Yuxing Lin**: investigation, software, formal analysis.

## Conflicts of Interest

The authors declare no conflicts of interest.

## Supporting information




**Supporting File**: advs77420‐sup‐0001‐SuppMat.docx.

## Data Availability

The data that support the findings of this study are available in the supplementary material of this article.
