# Supplementary material for: Lattice Chloride Shielding and Reconstructed Phosphate Armor for Stable Seawater Electrolysis
Source: Adv Sci (Weinh). 2026 Aug 25:e77420. Online ahead of print. doi: 10.1002/advs.77420 (PMC13505585; doi:10.1002/advs.77420)
Supplement: Supplementary file 1 [file ADVS-9999-e77420-s001.docx]

Supporting Information

**Lattice Chloride Shielding and Reconstructed Phosphate Armor for Stable Seawater Electrolysis**

Yatao Yan, Yuxing Lin, Xing Chen,* Shuo Yang, Ke Zhou, Jifang Chen,* Lubin Ni, and Ming Chen*

**1. Chemicals and Materials**

Cobaltous nitrate hexahydrate (Co(NO_3_)_2_·6H_2_O, 99%), Potassium nitrate (KNO_3_, 99%), Potassium fluoride (KF, 99%), Potassium chloride (KCl, 99%), Potassium bromide (KBr, 99%), and Potassium iodide (KI, 99%) were purchased from Aladdin. Titanium mesh (wire diameter: 0.32 mm) was obtained from Changsha Keliyuan. Sodium hypophosphite (NaH_2_PO_2_), Thiourea (CH_4_N_2_S), Sodium selenite (Na_2_SeO_3_, 97%), Hydrochloric acid (HCl, AR) and Potassium hydroxide (KOH, 85%) were purchased from Sinopharm Chemical Reagent Co. Pt/C (20 wt % Pt on Vulcan XC-72R), Iridium oxide (IrO_2_), and Nafion (5 wt %) were purchased from Sigma-Aldrich Chemical Reagent Co. All chemicals were used as received without further purification. Ultrapure water was used throughout the experiments.

**2. Synthesis of Catalysts**

All materials were synthesized via potentiostatic electrodeposition in a three-electrode system. All reagents were used without further purification. Titanium (Ti) mesh was employed as the cathodic substrate, with the geometric area controlled at 1 cm × 3 cm. Prior to electrodeposition, the Ti mesh was ultrasonically cleaned sequentially in 6 M concentrated hydrochloric acid, ultrapure water, and ethanol for 15 min each, followed by drying at room temperature before use. An Ag/AgCl electrode filled with saturated KCl solution and a graphite rod were used as the reference electrode and counter electrode, respectively.

**2.1 Co(OH)_2_ nanosheets**

The electrolyte was used to prepare the materials by adding 291.0 mg of Co(NO_3_)_2_·6H_2_O and 404.4 mg of KNO_3_ successively to 40 mL of deionized water, and ultrasonically stirred for several minutes until the solution was uniformly mixed. Electrodeposition experiments were performed under a constant potential of −0.8 V (vs. Ag/AgCl) and a deposition time of 90 min. The prepared catalysts were washed three times with water and ethanol, respectively, and dried overnight at 60 °C in a vacuum oven.

**2.2 X-Co(OH)_2_ (X = F, Cl, Br, I) nanosheets**

The synthesis procedure of X-Co(OH)_2_ was identical to that of Co(OH)_2_, except that KNO_3_ in the electrolyte was replaced with KF (232.4 mg), KCl (298.2 mg), KBr (476.0 mg), and KI (664.0 mg), respectively, while the electrodeposition parameters remained unchanged.

**2.3 Cl-Co(OH)_2_@CoP_4_ heterostructure**

72.75 mg of Co(NO_3_)_2_·6H_2_O and 1.0 g of NaH_2_PO_2_ were prepared into a 40 mL solution, sonicated for 10 min and stirred well to obtain the electrolyte. The Cl-Co(OH)_2_ obtained in the first step was used as the working electrode, and the voltage was set to be −0.6 V, and the time was set to be 30 min, 60 min, and 90 min, respectively, and the catalysts obtained corresponding to Cl-Co(OH)_2_@CoP_4_-30, Cl-Co(OH)_2_@CoP_4_-60, and Cl-Co(OH)_2_@CoP_4_-90. By replacing NaH_2_PO_2_ with thiourea (CH_4_N_2_S) and sodium selenite (Na_2_SeO_3_) respectively, with a deposition time of 60 minutes and other conditions remaining unchanged, catalysts Cl-Co(OH)_2_@Co-S and Cl-Co(OH)_2_@Co-Se can be obtained. Unless a specific comparison is being made, Cl-Co(OH)_2_@CoP_4_-60 is abbreviated to Cl-Co(OH)_2_@CoP_4_.

**2.4 F, Br, I-Co(OH)_2_@CoP_4_ heterostructure**

The synthesis procedure was identical to that of Cl-Co(OH)_2_@CoP_4_-60, except that F-Co(OH)_2_, Br-Co(OH)_2_, and I-Co(OH)_2_ were used as the working electrodes, respectively.

**3. Material characterization**

The characterization of morphology was conducted using Supra 55 SEM set at 5.0 kV, energy-dispersive X-ray spectroscopy (EDX, GENESIS, EDAX, USA). The transmission electron microscopy (TEM) images, high-resolution TEM (HRTEM) images, high-angle annular dark-field scanning TEM (HAADF-STEM), selected area electron diffraction (SAED), and energy-dispersive X-ray spectroscopic (EDS) elemental mappings were carried out on a Tecnai G2 F30 S-TWIN (FEI, USA) system using copper grids. X-ray diffraction (XRD) data was obtained with a graphite monochromator and Cu Kα radiation (λ= 0.1541 nm) on D8 advance superspeed powder diffractometer (Bruker). X-ray photoelectron spectroscopy (XPS, ESCALAB 250Xi, Thermo Scientific) was used to identify surface chemical species. The ultraviolet/visible (UV–vis) diffuse reflection spectra (DRS) were obtained on the Hitachi double-beam U-4100. The in-situ Raman spectra were collected using the Raman imaging microscope (Renishaw) with an excitation wavelength of 532 nm. The UV-Vis spectrophotometer (UV2550, Shimadzu, Japan) was used to determine the hypochlorite concentration during the reaction process.

**4. Electrochemical measurement**

All three-electrode electrochemical tests were carried out on a CHI 760E electrochemical workstation. The self-supported catalytic electrodes were used as the working electrode (WE), while a platinum plate and Hg/HgO electrode were applied as the counter electrode (CE) and reference electrode (RE), respectively. The test potentials were calculated by E(RHE) = E(Hg/HgO) + (0.098 + 0.0592 pH) V with respect to the reverse hydrogen electrode (RHE). Unless otherwise specified, all materials were subjected to 90% iR compensation during the polarization curve measurements, with a scan rate of 5 mV s^−1^. Tafel curves were built from *η* = a + blog |*j*| (*η*, a, and b referred to overpotential, electron transfer coefficient, and Tafel slope, respectively).^[1]^ The linear relationship of potentials against current density was fitted to obtain the Tafel slope. Cyclic voltammetry (CV) tests were used to determine the bilayer capacitance and electrochemical active area of the self-supported catalysts. The potential window of CV was 0.8–0.9 V vs RHE. To measure double-layer capacitance (*C*_dl_) value, CV curves were performed in the non-Faradaic regions at scan rates of 20, 40, 60, 80, and 100 mV s^−1^. The *C*_dl_ was estimated by plotting the ∆*j*/2 = (*j*_a_‒*j*_c_)/2 at 0.85 V vs RHE against the scan rate, where *j*_a_ (A cm^−2^) is the anode current density at open circuit potential, *j*_c_ (A cm^−2^) is the cathode current density and *v* is the scanning rate.^[2,3]^ The electrochemical surface area (ECSA) values were calculated by the electrochemical double layer capacitance. The ECSA was calculated ECSA=*C*_dl_/*C*_s_, where *C*_s_ is the specific capacitance using an average value of 0.040 mF cm^−2^ referred to literature.^[4-6]^ The electrochemical impedance spectroscopy (EIS) measurement was conducted at potential corresponding to 10 mA cm^−2^ for HER and OER processes, in the frequency range of 10^5^ Hz to 0.1 Hz with an amplitude of 10 mV. Long-term stability was evaluated by recording the current–time curves of the catalysts.

**5. Theoretical Calculations**

The Vienna Ab Initio Simulation Package (VASP) is a software package used for performing first-principles density functional theory (DFT) calculations.^[7]^ The interaction between ions and electrons was described by the projector augmented-wave (PAW) method.^[8]^ The exchange-correlation interactions were treated within the generalized gradient approximation (GGA) using the Perdew-Burke-Ernzerhof (PBE) functional.^[9,10]^ Total energy calculations employ the projected augmented wave (PAW) method and utilize a plane-wave basis set with an energy cutoff of 450 eV. Considering the strong electron correlation of Co 3d orbitals, the DFT+U method was applied, with an effective Hubbard U value of 3.3 eV for Co atoms. The convergence criteria for forces and energy are set to 0.02 eV Å^−1^ and 10^−5^ eV, respectively.

For structural modeling, the Co(OH)_2_ (001) surface was selected as the substrate because it represents the dominant exposed surface of layered cobalt hydroxide. A 3 × 3 × 1 supercell of Co(OH)_2_ (001) was constructed to provide sufficient surface area for interfacial coupling and adsorbate optimization. The CoP_4_ (111) surface was selected to construct the surface phosphide component. The Co(OH)_2_@CoP_4_ heterostructure was then built by integrating the CoP_4_ (111) slab with the Co(OH)_2_ (001) slab. For the Cl-Co(OH)_2_@CoP_4_ model, one lattice site in the Co(OH)_2_ framework was substituted/modified by Cl to simulate lattice Cl incorporation. A vacuum layer of 15 Å was introduced along the z direction to avoid interactions between periodic images. During structural optimization, the Brillouin zone was sampled using a 3 × 3 × 1 Monkhorst-Pack k-point mesh, while a denser 5 × 5 × 1 k-point mesh was used for electronic structure calculations.

For the oxygen evolution reaction, the four-electron associative mechanism in alkaline media was considered. The elementary steps are described as follows:

|  | ∗ + OH^-^ → ^∗^OH + e^-^ | (1) |
| --- | --- | --- |
|  | ^∗^OH + OH^-^ → ^*^O + H_2_O + e^-^ | (2) |
|  | ^*^O + OH^-^ → ^*^OOH + e^-^ | (3) |
|  | ^*^OOH + OH^-^ → ∗ + O_2_ + H_2_O + e^-^ | (4) |

The Gibbs free energy change of each elementary step was calculated according to the computational hydrogen electrode model by including zero-point energy correction, entropy correction, applied electrode potential, and pH correction:

|  | Δ*G* = Δ*E* + Δ*E*_ZPE_ – TΔ*S* – ne*U* + *k*_B_*T*ln10 × pH | (5) |
| --- | --- | --- |

where Δ*E* is the reaction energy obtained from DFT calculations, Δ*E*_ZPE_ is the zero-point energy correction, TΔ*S* is the entropy correction, n is the number of transferred electrons, e is the elementary charge, *U* is the applied electrode potential versus RHE, *k*_B_ is the Boltzmann constant, and pH represents the electrolyte pH value. For the OER process, each elementary step involves one electron transfer; therefore, the free energy of each step is corrected by −eU. The pH correction term was included to account for the alkaline reaction environment. The theoretical OER overpotential was calculated according to:

|  | *η*_OER_ = [max(Δ*G*_1_, Δ*G*_2_, Δ*G*_3_, Δ*G*_4_)]/e − 1.23 | (6) |
| --- | --- | --- |

**Figure S1.** SEM images of (a, b) pure Titanium mesh and (c, d) Cl-Co(OH)_2_ nanosheets.

**Figure S2.** (a-b) SEM images of Co(OH)_2_ nanosheets.

**Figure S3.** TEM images of (a, b) Co(OH)_2_ and (c, d) Cl-Co(OH)_2_ nanosheets.

**Figure S4.** (a-b) SEM images and (e-h) element mapping for Cl-Co(OH)_2_ corresponding to different KCl concentrations of 0.05 M, 0.10 M, 0.15 M, and 0.20 M, respectively.

**Figure S5.** The energy-dispersive X-ray elemental analysis and atomic percent of Cl-Co(OH)_2_@CoP_4_.

**Figure S6.** (a, c, e, g) SEM images and (b, d, f, h) element mapping for F-Co(OH)_2_, Cl-Co(OH)_2_, Br-Co(OH)_2_, I-Co(OH)_2_, respectively.

**Figure S7.** SEM images of (a) F-Co(OH)_2_@CoP_4_, (b) Cl-Co(OH)_2_@CoP_4_, (c) Br-Co(OH)_2_@CoP_4_, (d) I-Co(OH)_2_@CoP_4_.

**Figure S8.** XPS survey of Co(OH)_2_, Cl-Co(OH)_2_, and Cl-Co(OH)_2_@CoP_4_.

**Figure S9.** Calculated work functions of (a) CoP_4_, (b) Co(OH)_2_, (c) Cl-Co(OH)_2_, and (d) Cl-Co(OH)_2_@CoP_4_.

**Figure S10.** (a) The OER polarization curves and (b) Tafel plots of Co(OH)_2_ and Cl- Co(OH)_2_ in 1.0 M KOH.

**Figure S11.** Analysis of the alkaline OER activity of Cl-Co(OH)_2_ prepared at different KCl concentrations: (a) LSV curves and (b) Tafel plots.

**Figure S12.** (a) The OER polarization curves of F, Cl, Br, I-Co(OH)_2_ in 1.0 M KOH, (b) corresponding Tafel plots, (c) the overpotential of catalysts at different current densities.

**Figure S13.** OER performance in 1.0 M KOH: (a) LSV curves, (b) Tafel plots and (c) overpotentials at different current densities for different catalysts.

**Figure S14.** Performance comparison of Cl-Co(OH)_2_@CoP_4_-60 with reported excellent catalysts for OER at 50 mA cm^−2^ in 1 M KOH.

**Figure S15.** (a) Analogue circuit diagrams and (b) Nyquist plots of Cl-Co(OH)_2_@CoP_4_-30, 60 and 90 at 10 mA cm^−2^ for OER in 1 M KOH.

**Figure S16.** Cyclic voltammetric profiles of (a) Cl-Co(OH)_2_@CoP_4_-30, (b) Cl-Co(OH)_2_@CoP_4_-60 and (c) Cl-Co(OH)_2_@CoP_4_-90 measured at different scan rates (20 mV s^−1^ ~ 100 mV s^−1^) after OER activation in 1 M KOH. (d) The double layer capacitance of Cl-Co(OH)_2_@CoP_4_-30, 60 and 90.

**Figure S17.** Electrochemical surface area (ECSA) of Cl-Co(OH)_2_@CoP_4_-30, 60 and 90 were also determined according to the CV curves.

**Figure S18.** (a) The OER polarization curves and (b) Tafel plots of Co(OH)_2_ and Cl- Co(OH)_2_ in alkaline seawater.

**Figure S19.** (a) The OER polarization curves and (b) Tafel plots of F, Cl, Br, I-Co(OH)_2_ in alkaline seawater.

**Figure S20.** (a) Polarization curves of Cl-Co(OH)_2_@CoP_4_ electrode for OER in different electrolytes. (b) Relationship between overpotential and NaCl concentration in the electrolyte.

**Figure S21.** (a) The OER polarization curves and (b) Tafel plots of F, Cl, Br, I-Co(OH)_2_@CoP_4_ in alkaline seawater.

**Figure S22.** (a-d) Cyclic voltammetric profiles of F, Cl, Br, I-Co(OH)_2_@CoP_4_ measured at different scan rates (20 mV s^−1^ ~ 100 mV s^−1^) after OER activation in alkaline seawater.

**Figure S23.** (a) The double layer capacitance and (b) Corresponding ECSA of F, Cl, Br, I-Co(OH)_2_@CoP_4_.

**Figure S24.** Nyquist plots of F, Cl, Br, I-Co(OH)_2_@CoP_4_ at 50 mA cm^−2^ for OER in alkaline seawater.

**Figure S25.** (a) Polarization curves of Cl-Co(OH)_2_@CoP_4_ for the OER before and after 5000 CV cycles in alkaline seawater. (b) *I*-*t* test at different current densities of Cl-Co(OH)_2_@CoP_4_-60 for OER in alkaline seawater.

**Figure S26.** SEM images of (a, b) Cl-Co(OH)_2_@Co-S and (c, d) Cl-Co(OH)_2_@Co-Se.

**Figure S27.** XRD pattern of Cl-Co(OH)_2_@Co-S.

**Figure S28.** (a) SEM image of the cross section and (b-d) element mapping for Cl-Co(OH)_2_@Co-S.

**Figure S29.** XRD pattern of Cl-Co(OH)_2_@Co-Se.

**Figure S30.** (a) SEM image of the cross section and (b-d) element mapping for Cl-Co(OH)_2_@Co-Se.

**Figure S31.** (a) The OER polarization curves of Cl-Co(OH)_2_ and Cl-Co(OH)_2_@Co-P, S, Se in 1.0 M KOH, (b) corresponding Tafel plots.

**Figure S32.** (a) The OER polarization curves of Cl-Co(OH)_2_ and Cl-Co(OH)_2_@Co-P, S, Se in alkaline seawater, (b) corresponding Tafel plots.

**Figure S33.** HER performance in alkaline seawater: (a) LSV curves, (b) Tafel plots and (c) overpotentials at different current densities for different catalysts.

**Figure S34.** (a) The HER polarization curves and (b) Tafel plots of F, Cl, Br, I-Co(OH)_2_@CoP_4_ in alkaline seawater.

**Figure S35.** Nyquist plots of F, Cl, Br, I-Co(OH)_2_@CoP_4_ at 50 mA cm^−2^ for HER in alkaline seawater.

**Figure S36.** (a) Polarization curves of Cl-Co(OH)_2_@CoP_4_ for the HER before and after 5000 CV cycles in alkaline seawater. (b) *I*-*t* test at different current densities of Cl-Co(OH)_2_@CoP_4_-60 for HER in alkaline seawater.

**Figure S37.** The amount and Faradaic efficiency of H_2_ and O_2_ varying with time in alkaline seawater.

**Figure S38.** The cell voltage comparison of Cl-Co(OH)_2_@CoP_4_ with recently reported electrocatalysts at current density of 100 and 200 mA cm^−2^ in alkaline seawater.

**Figure S39.** XRD patterns of Cl-Co(OH)_2_@CoP_4_-60 after stability test.

**Figure S40.** (a, b) SEM images of Cl-Co(OH)_2_@CoP_4_ after stability test for OER in 1.0 M KOH. (c, d) SEM images of Cl-Co(OH)_2_@CoP_4_ after stability test for OER in alkaline seawater.

**Figure S41.** (a, b) TEM, (c) HRTEM images, and (d) SAED pattern of Cl-Co(OH)_2_@CoP_4_ after stability test in alkaline seawater.

**Figure S42.** The high-resolution spectra of (a) Co 2p, (b) P 2p and (c) O 1s of Cl-Co(OH)_2_@CoP_4_ after stability test for anode in 1.0 M KOH and alkaline seawater.

**Figure S43.** (a, b) SEM images of Cl-Co(OH)_2_@CoP_4_ after stability test for HER in 1.0 M KOH. (c, d) SEM images of Cl-Co(OH)_2_@CoP_4_ after stability test for HER in alkaline seawater.

**Figure S44.** The high-resolution spectra of (a) Co 2p, (b) P 2p and (c) O 1s of Cl-Co(OH)_2_@CoP_4_ after stability test for cathode in 1.0 M KOH and alkaline seawater.

**Figure S45.** The Raman spectra of Cl-Co(OH)_2_ and Cl-Co(OH)_2_@CoP_4_.

**Figure S46.** The theoretical models of CoP_4_, Co(OH)_2_@CoP_4_ and X-Co(OH)_2_@CoP_4_ (X=F, Cl, Br, I).

**Figure S47.** The free energy diagrams for HER on CoP_4_, Co(OH)_2_@CoP_4_ and Cl-Co(OH)_2_@CoP_4_.

**Figure S48.** The Projected density of states of CoP_4_, Co(OH)_2_@CoP_4_ and Cl-Co(OH)_2_@CoP_4_.

**Figure S49.** Gibbs free energy diagrams of CoP_4_, Co(OH)_2_@CoP_4_ and Cl-Co(OH)_2_@CoP_4_ for OER process at U = 0 V.

**Figure S50.** Gibbs free energy diagrams of F, Cl, Br, I-Co(OH)_2_@CoP_4_ for OER process at U = 0 V.

**Figure S51.** Reaction pathway diagram for the four steps of the OER on F-Co(OH)_2_@CoP_4_.

**Figure S52.** Reaction pathway diagram for the four steps of the OER on Br-Co(OH)_2_@CoP_4_.

**Figure S53.** Reaction pathway diagram for the four steps of the OER on I-Co(OH)_2_@CoP_4_.

**Figure S54.** Chlorine chemistry on Co(OH)_2_@CoP_4_ and Cl-Co(OH)_2_@CoP_4_ in alkaline seawater electrolyte (1.0 M KOH + real seawater). (a, b) The standard curve of different molars of NaClO after reaction with KI. (c) UV–vis absorption curves of ClO^−^ products over Cl-Co(OH)_2_@CoP_4_ at different reaction potentials, and (d) the corresponding FE of O_2_ and ClO^−^. (e) The corresponding UV-vis curves of Co(OH)_2_@CoP_4_ and Cl-Co(OH)_2_@CoP_4_ reaction for 50 hours. (f) The corresponding FE of O_2_ and ClO^−^ and content of ClO^−^ for different electrolytes after long-term reaction with the excessive KI.

**Figure S55.** Comparison of the operating voltages of advanced AEM seawater electrolyzers at an industrial-level current density of 500 mA cm^−2^.

**Table S1.** XPS spectrum fitting for peak position of Co(OH)_2_, Cl-Co(OH)_2_ and Cl-Co(OH)_2_@CoP_4_.

| Samples | Co 2p_3/2_ | | | | |  |
| --- | --- | --- | --- | --- | --- | --- |
|  | Co^2+^ (eV) | Co^3+^ (eV) | Sat. 1 (eV) | Sat. 2 (eV) | Co^2+^/Co^3+^ | |
| Co(OH)_2_ | 782.76 | 780.71 | 786.73 | 790.38 | 0.61 | |
| Cl-Co(OH)_2_ | 782.50 | 780.40 | 785.48 | 789.81 | 0.74 | |
| Cl-Co(OH)_2_@CoP_4_ | 782.23 | 780.22 | 786.88 |  | 0.51 | |

**Table S2.** Comparison of the OER activity of Cl-Co(OH)_2_@CoP_4_ with recently reported electrocatalysts in alkaline electrolyte.

| Catalyst | Electrolyte | *j* (mA cm^−2^) | *η* (mV) | Tafel slope (mV dec^−1^) | Ref. |
| --- | --- | --- | --- | --- | --- |
| Ru/d-NiFe LDH | 1.0 M KOH | 50 | 283 | 60.48 | ^[11]^ |
| FeOOH/Co(OH)_2_ | 1.0 M KOH | 50 | 265 | 38 | ^[12]^ |
| N-doped CoO | 1.0 M KOH | 50 | ~320 | 75 | ^[13]^ |
| Ni-MOF/LDH | 1.0 M KOH | 50 | 271 | 36 | ^[14]^ |
| NiCo-LDH-OH | 1.0 M KOH | 50 | ~368 | 115 | ^[15]^ |
| Ru-S-NiFe LDH | 1.0 M KOH | 50 | ~259 | 81.65 | ^[16]^ |
| F,N-NiFe LDH | 1.0 M KOH | 50 | ~245 | 30.0 | ^[17]^ |
| NiMoO_4_@CoFe-LDH | 1.0 M KOH | 50 | ~233 | 49.6 | ^[18]^ |
| NiCo_2_O_4_-F_1_ | 1.0 M KOH | 50 | 349 | 96.0 | ^[19]^ |
| NiFe-LDH/Ni_4_Mo | 1.0 M KOH | 50 | ~249 | 42.0 | ^[20]^ |
| Cl-Co(OH)_2_@CoP_4_ | 1.0 M KOH | 50 | 252 | 100.6 | This work |

**Table S3.** Comparison of the HER activity of Cl-Co(OH)_2_@CoP_4_ with recently reported electrocatalysts in alkaline electrolyte.

| Catalyst | Electrolyte | *j* (mA cm^-2^) | *η* (mV) | Tafel slope (mV dec^-1^) | Ref. |
| --- | --- | --- | --- | --- | --- |
| Ru/d-NiFe LDH | 1.0 M KOH | 100 | 145 | 50.4 | ^[11]^ |
| Mn-NiCoP | 1.0 M KOH | 100 | 148 | 84 | ^[21]^ |
| Ru-Ni_3_N/NiO | 1.0 M KOH | 100 | 97 | 58 | ^[22]^ |
| Ni_1.0_Cu_1.0_DC | 1.0 M KOH | 100 | 216 | 42 | ^[23]^ |
| NiIr_SAA_-NiFe-LDH | 1.0 M KOH | 200 | 400 | 32.9 | ^[24]^ |
| Se-FeCo-LDH | 1.0 M KOH | 100 | 283 | 113.5 | ^[25]^ |
| Ni_2_P/CoP-40 | 1.0 M KOH | 100 | 152 | 63.9 | ^[26]^ |
| CNFMPO | 1.0 M KOH | 100 | 225 | 33.5 | ^[27]^ |
| (FeCoNi)OH-S/NF | 1.0 M KOH | 100 | 278 | -- | ^[28]^ |
| PdIr BNRs | 1.0 M KOH | 25 | 123 | 117 | ^[29]^ |
| NiMoO_4_@NiFeP | 1.0 M KOH | 100 | 188 | 89.3 | ^[30]^ |
| NiFeS/NF | 1.0 M KOH | 100 | 196 | 102.9 | ^[31]^ |
| Cl-Co(OH)_2_@CoP_4_ | 1.0 M KOH | 100 | 123 | 70.8 | This work |

**Table S4.** Overall water splitting performance comparison of Cl-Co(OH)_2_@CoP_4_ with recently reported catalysts in 1 M KOH seawater.

| Catalyst | Electrolyte | *j* (mA cm^-2^) | *E* (V) | Ref. |
| --- | --- | --- | --- | --- |
| NiMoO_4_@NiFeP | 1.0 M KOH seawater | 100 | 1.71 | ^[30]^ |
| Ru@FeCo–Ni(OH)_2_ | 1.0 M KOH seawater | 100 | 1.63 | ^[32]^ |
| Co-Fe_2_P | 1.0 M KOH seawater | 100 | 1.69 | ^[33]^ |
| NiMoN//S-(Ni,Fe)OOH | 1.0 M KOH seawater | 100 | 1.661 | ^[34]^ |
| CeNiFe@CuO//Pt | 1.0 M KOH seawater | 100 | ~1.92 | ^[35]^ |
| (FeCoNi)OH-S/NF | 1.0 M KOH seawater | 100 | 1.9 | ^[28]^ |
| NiFeS/NF | 1.0 M KOH seawater | 100 | 1.685 | ^[31]^ |
| FeOOH_0.6_/Ni(HCO_3_)_2_//Pt/C | 1.0 M KOH seawater | 100 | 1.554 | ^[36]^ |
| Ni-MoN//SSM | 1.0 M KOH seawater | 100 | 1.62 | ^[37]^ |
| NiFeLDH//NiS_x_ | 1.0 M KOH seawater | 200 | ~1.95 | ^[38]^ |
| NiCoS@NiMoS | 1.0 M KOH seawater | 200 | 1.81 | ^[39]^ |
| NiCo-LDH//NiMoN | 1.0 M KOH seawater | 200 | 1.7 | ^[40]^ |
| Pt/C/NF\|\|Ce-NiFe LDH/NF | 1.0 M KOH seawater | 200 | 2.07 | ^[41]^ |
| Co_3-x_Pd_x_O_4_ | 1.0 M KOH seawater | 200 | 1.78 | ^[42]^ |
| S,P-(Ni,Mo,Fe)OOH/NiMoP/wood | 1.0 M KOH seawater | 200 | 1.805 | ^[43]^ |
| Cl-Co(OH)_2_@CoP_4_ | 1.0 M KOH seawater | 100/200 | 1.578/1.595 | This work |

**Table S5.** Operation voltages of Cl-Co(OH)_2_@CoP_4_ and recently reported catalysts in different electrolytes.

| Anode | Cathode | Electrolyte | *j* (A cm^-2^) | *E* (V) | Ref. |
| --- | --- | --- | --- | --- | --- |
| NiFe LDH | RuMo/Cu_2_O@C | 1.0 M KOH + seawater | 0.5 | ~1.75 | ^[44]^ |
| SO_4_^2−^/CoFe LDH | Pt/C | 1.0 M KOH + seawater | 0.5 | ~2.25 | ^[45]^ |
| FeMoOOH/NF | Co_2_Mo_3_O_8_/MoO_2_ | 1.0 M KOH + seawater | 0.5 | ~1.96 | ^[46]^ |
| CoFe LDH | Pt/C | 1.0 M KOH + seawater | 0.5 | 2.15 | ^[47]^ |
| Ir/CoFe-LDH | Pt/CoP | 1.0 M KOH+0.5 M NaCl | 0.5 | 1.63 | ^[48]^ |
| NiFe LDH | NiS_x_ | 30% KOH + seawater | 0.4 | ~1.96 | ^[38]^ |
| Os-Ni_4_Mo/MoO_2_ | Os-Ni_4_Mo/MoO_2_ | 1.0 M KOH + seawater | 0.5 | ~3.5 | ^[49]^ |
| NiFeCoP | Pt/C | 1.0 M KOH + seawater | 0.5 | 2.4 | ^[50]^ |
| CrO_4_^2–^-NiFe LDH/Cr_2_O_3_/NF | Pt/C | 1.0 M KOH + seawater | 0.5 | 2.4 | ^[51]^ |
| NiFe LDH/NF | Pt-Ni_3_N@V_2_O_3_/NF | 1.0 M KOH + seawater | 0.5 | 1.89 | ^[52]^ |
| Ni_3_FeN@PO_4_^3−^/NF | Pt-Ni@NiMoN/NF | 1.0 M KOH + seawater | 0.5 | 1.95 | ^[53]^ |
| Cl-Co(OH)_2_@CoP_4_ | Cl-Co(OH)_2_@CoP_4_ | 1.0 M KOH + seawater | 0.5 | 1.67 | This work |

References

[1] J. Ren, Z. Hu, C. Chen, Y. Liu, Z. Yuan, *J. Energy Chem.* **2017**, *26*, 1196-1202.
[2] C. Guan, W. Xiao, H.J. Wu, X.M. Liu, W.J. Zang, H. Zhang, J. Ding, Y.P. Feng, S.J. Pennycook, J. Wang, *Nano Energy* **2018**, *48*, 73-80.
[3] K. Li, J. Zhang, R. Wu, Y. Yu, B. Zhang, *Adv. Sci.* **2016**, *3*, 1500426.
[4] H. Xu, H. Jia, B. Fei, Y. Ha, H. Li, Y. Guo, M. Liu, R. Wu, *Appl. Catal. B* **2020**, *268*, 118404.
[5] H.Q. Song, J.K. Yu, Z.Y. Tang, B. Yang, S.Y. Lu, *Adv. Energy Mater.* **2022**, *12*, 2102573.
[6] Y. Lu, B. Li, N. Xu, Z. Zhou, Y. Xiao, Y. Jiang, T. Li, S. Hu, Y. Gong, Y. Cao, *Nat. Commun.* **2023**, *14*, 6965.
[7] G. Kresse, J. Hafner, *Phys. Rev. B* **1993**, *48*, 13115-13118.
[8] P.E. Blochl, *Phys. Rev. B* **1994**, *50*, 17953-17979.
[9] S. Grimme, *J. Comput. Chem.* **2006**, *27*, 1787-99.
[10] J.P. Perdew, K. Burke, M. Ernzerhof, *Phys. Rev. Lett.* **1996**, *77*, 3865-3868.
[11] Y. Chen, Y. Liu, W. Zhai, H. Liu, T. Sakthivel, S. Guo, Z. Dai, *Adv. Energy Mater.* **2024**, *14*, 2400059.
[12] D. Li, R. Xiang, F. Yu, J. Zeng, Y. Zhang, W. Zhou, L. Liao, Y. Zhang, D. Tang, H. Zhou, *Adv. Mater.* **2023**, *36*, 2305685.
[13] P.C. Yu, X.L. Zhang, T.Y. Zhang, X.Y. Tao, Y. Yang, Y.H. Wang, S.C. Zhang, F.Y. Gao, Z.Z. Niu, M.H. Fan, M.R. Gao, *J. Am. Chem. Soc.* **2024**, *146*, 20379-20390.
[14] W.-D. Zhang, Q.-T. Hu, L.-L. Wang, J. Gao, H.-Y. Zhu, X. Yan, Z.-G. Gu, *Appl. Catal. B* **2021**, *286*, 119906.
[15] H. Yang, Z. Zhou, H. Yu, H. Wen, R. Yang, S. Peng, M. Sun, L. Yu, *J. Colloid Interface Sci.* **2023**, *636*, 11-20.
[16] Y. Zhu, J. Wang, G. Weiser, M. Klingenhof, T. Koketsu, S. Liu, Y. Pi, G. Henkelman, X. Shi, J. Li, C.W. Pao, M.H. Yeh, W.H. Huang, P. Strasser, J. Ma, *Adv. Energy Mater.* **2025**, *15*, 2500554.
[17] Q. Yang, Y. Li, A. Kong, Y. Li, T. An, C. Yang, Y. Zhao, L. Lai, C. Feng, X. Zhou, P. Chen, K. Pan, Z. Wang, H. Huang, F. Yu, *Appl. Catal. B* **2026**, *383*, 126130.
[18] Y. Yin, S. Wang, B. Chen, A. Zhang, L. Wang, F. Bai, T. Wang, J. Zhang, Z. Che, *Small* **2025**, *21*, e08103.
[19] Y. Yue, X. Zhong, M. Sun, J. Du, W. Gao, W. Hu, C. Zhao, J. Li, B. Huang, Z. Li, C. Li, *Adv. Mater.* **2025**, *37*, e2418058.
[20] F. Wu, F. Tian, M. Li, S. Geng, L. Qiu, L. He, L. Li, Z. Chen, Y. Yu, W. Yang, Y. Hou, *Angew. Chem. Int. Ed.* **2025**, *64*, e202413250.
[21] G. Ma, J. Ye, M. Qin, T. Sun, W. Tan, Z. Fan, L. Huang, X. Xin, *Nano Energy* **2023**, *115*, 108679.
[22] R. Liu, M. Sun, X. Liu, Z. Lv, X. Yu, J. Wang, Y. Liu, L. Li, X. Feng, W. Yang, B. Huang, B. Wang, *Angew. Chem. Int. Ed.* **2023**, *62*, e202312644
[23] A. Kumar, S.K. Purkayastha, A.K. Guha, M.R. Das, S. Deka, *ACS Catal.* **2023**, *13*, 10615-10626.
[24] Y. Hu, T. Shen, Z. Song, Z. Wu, S. Bai, G. Liu, X. Sun, Y. Wang, S. Hu, L. Zheng, Y.-F. Song, *ACS Catal.* **2023**, *13*, 11195-11203.
[25] Y. Gong, H. Zhao, Y. Sun, D. Xu, D. Ye, Y. Tang, T. He, J. Zhang, *J. Colloid Interface Sci.* **2023**, *650*, 636-647.
[26] Y. Tan, J. Feng, L. Kang, L. Liu, F. Zhao, S. Zhao, D.J.L. Brett, P.R. Shearing, G. He, I.P. Parkin, *Energy Environ. Sci.* **2022**, *6*, e12398.
[27] H.-M. Zhang, L. Zuo, Y. Gao, J. Guo, C. Zhu, J. Xu, J. Sun, *J. Mater. Sci. Technol.* **2024**, *173*, 1-10.
[28] Y. Yang, M. Lin, Y. Wu, R. Chen, D. Guo, L. Liu, *J. Colloid Interface Sci.* **2023**, *647*, 510-518.
[29] S. Liu, H. Zhang, H. Yu, K. Deng, Z. Wang, Y. Xu, L. Wang, H. Wang, *Small* **2023**, *19*, 2300388.
[30] D. Guo, Z. Zhao, M.-Y. Zong, C. Fan, W. Zheng, D. Wang, *ACS Sustainable Chem. Eng.* **2023**, *11*, 8362-8373.
[31] J. Chen, L. Zhang, J. Li, X. He, Y. Zheng, S. Sun, X. Fang, D. Zheng, Y. Luo, Y. Wang, J. Zhang, L. Xie, Z. Cai, Y. Sun, A.A. Alshehri, Q. Kong, C. Tang, X. Sun, *J. Mater. Chem. A* **2023**, *11*, 1116-1122.
[32] J. Lu, Z. Yu, X. Wei, X. Zhang, X. Wang, K. Liu, Y. Cai, H. Pan, D. Liu, Z. Wang, *Adv. Mater.* **2026**, *38*, e15156.
[33] S. Wang, P. Yang, X. Sun, H. Xing, J. Hu, P. Chen, Z. Cui, W. Zhu, Z. Ma, *Appl. Catal. B* **2021**, *297*, 120386.
[34] L. Yu, L. Wu, B. McElhenny, S. Song, D. Luo, F. Zhang, Y. Yu, S. Chen, Z. Ren, *Energy Environ. Sci.* **2020**, *13*, 3439-3446.
[35] H. Zhao, K. Deng, X. Liu, P. Liu, X. Lv, W. Tian, J. Ji, *Adv. Funct. Mater.* **2025**, *35*, 2508539.
[36] M. Lin, Y. Yang, Y. Song, D. Guo, L. Yang, L. Liu, *Nano Research* **2022**, *16*, 2094-2101.
[37] L. Wu, F. Zhang, S. Song, M. Ning, Q. Zhu, J. Zhou, G. Gao, Z. Chen, Q. Zhou, X. Xing, T. Tong, Y. Yao, J. Bao, L. Yu, S. Chen, Z. Ren, *Adv. Mater.* **2022**, *34*, e2201774.
[38] H. Liu, W. Shen, H. Jin, J. Xu, P. Xi, J. Dong, Y. Zheng, S.Z. Qiao, *Angew. Chem. Int. Ed.* **2023**, *62*, e202311674.
[39] C. Wang, M. Zhu, Z. Cao, P. Zhu, Y. Cao, X. Xu, C. Xu, Z. Yin, *Appl. Catal. B* **2021**, *291*, 120071.
[40] S. Zhou, J. Wang, J. Li, L. Fan, Z. Liu, J. Shi, W. Cai, *Appl. Catal. B* **2023**, *332*, 122749.
[41] Y. Yao, S. Sun, H. Zhang, Z. Li, C. Yang, Z. Cai, X. He, K. Dong, Y. Luo, Y. Wang, Y. Ren, Q. Liu, D. Zheng, W. Zhuang, B. Tang, X. Sun, W. Hu, *J. Energy Chem.* **2024**, *91*, 306-312.
[42] N. Wang, P. Ou, S.F. Hung, J.E. Huang, A. Ozden, J. Abed, I. Grigioni, C. Chen, R.K. Miao, Y. Yan, J. Zhang, Z. Wang, R. Dorakhan, A. Badreldin, A. Abdel‐Wahab, D. Sinton, Y. Liu, H. Liang, E.H. Sargent, *Adv. Mater.* **2023**, *35*, 2210057.
[43] H. Chen, Y. Zou, J. Li, K. Zhang, Y. Xia, B. Hui, D. Yang, *Appl. Catal. B* **2021**, *293*, 120215.
[44] X. Yang, H. Shen, X. Xiao, Z. Li, H. Liang, S. Chen, Y. Sun, B. Jiang, G. Wen, S. Wang, L. Zhang, *Adv. Mater.* **2025**, *37*, e2416658.
[45] Y. Yu, W. Zhou, X. Zhou, J. Yuan, X. Zhang, X. Meng, F. Sun, J. Gao, G. Zhao, *Adv. Funct. Mater.* **2025**, *35*, 2419871.
[46] J. Sun, S. Zhou, Z. Zhao, S. Qin, X. Meng, C.-H. Tung, L.-Z. Wu, *Energy Environ. Sci.* **2025**, *18*, 1952-1962.
[47] Y. Yu, W. Zhou, J. Yuan, X. Zhou, X. Meng, X. Zhang, X. Li, N. Xue, Y. Chen, X. Xia, M. Gu, J. Chen, X. Wang, F. Sun, J. Gao, G. Zhao, *Energy Environ. Sci.* **2025**, *18*, 9949-9958.
[48] W. Liu, X. Guo, Z. Jiang, J. Yu, L. Zhou, T. Li, Y. Guo, S. Li, B. Ding, K. Wang, Y. Yang, H. Xin, D. Zhou, Y. Kuang, X. Sun, *Adv. Energy Mater.* **2024**, *15*, 2404978.
[49] D. Liu, X. Wei, J. Lu, X. Wang, K. Liu, Y. Cai, Y. Qi, L. Wang, H. Ai, Z. Wang, *Adv. Mater.* **2024**, *36*, e2408982.
[50] Y. Yu, W. Zhou, X. Zhou, J. Yuan, X. Zhang, L. Wang, J. Li, X. Meng, F. Sun, J. Gao, G. Zhao, *ACS Catal.* **2024**, *14*, 18322-18332.
[51] Z. Cai, J. Liang, Z. Li, T. Yan, C. Yang, S. Sun, M. Yue, X. Liu, T. Xie, Y. Wang, T. Li, Y. Luo, D. Zheng, Q. Liu, J. Zhao, X. Sun, B. Tang, *Nat. Commun.* **2024**, *15*, 6624.
[52] H. Hu, Z. Zhang, L. Liu, X. Che, J. Wang, Y. Zhu, J.P. Attfield, M. Yang, *Sci. Adv.* **2024**, *10*, eadn7012.
[53] H. Hu, X. Wang, Z. Zhang, J. Liu, X. Yan, X. Wang, J. Wang, J.P. Attfield, M. Yang, *Adv. Mater.* **2025**, *37*, e2415421.
